# Characterization of sediment microbial communities at two sites with low hydrocarbon pollution in the southeast Gulf of Mexico

**DOI:** 10.7717/peerj.10339

**Published:** 2020-12-08

**Authors:** Pablo Suárez-Moo, Araceli Lamelas, Itza Garcia-Bautista, Luis Felipe Barahona-Pérez, Gloria Sandoval-Flores, David Valdes-Lozano, Tanit Toledano-Thompson, Erik Polanco-Lugo, Ruby Valdez-Ojeda

**Affiliations:** 1 Red de Estudios Moleculares Avanzados, Instituto de Ecología, Xalapa, Veracruz, Mexico; 2 Unidad de Energia Renovable, Centro de Investigacion Cientifica de Yucatan, Merida, Yucatan, Mexico; 3 Unidad Académica Multidisciplinaria Reynosa-Aztlán, Universidad Autonoma de Tamaulipas, Merida, Yucatan, Mexico; 4 Centro de Investigación y de Estudios Avanzados, Insituto Politecnico Nacional, Merida, Yucatan, Mexico; 5 Campus de Ciencias Biológicas y Agropecuarias,, Universidad Autonoma de Yucatan, Merida, Yucatan, Mexico

**Keywords:** Bacteria, Hydrocarbon pollution, Southeast gulf of mexico, Yucatan, Marine sediments

## Abstract

**Background:**

Coastal ecosystems are prone to hydrocarbon pollution due to human activities, and this issue has a tremendous impact on the environment, socioeconomic consequences, and represents a hazard to humans. Bioremediation relies on the ability of bacteria to metabolize hydrocarbons with the aim of cleaning up polluted sites.

**Methods:**

The potential of naturally occurring microbial communities as oil degraders was investigated in Sisal and Progreso, two port locations in the southeast Gulf of Mexico, both with a low level of hydrocarbon pollution. To do so, we determined the diversity and composition of bacterial communities in the marine sediment during the dry and rainy seasons using 16S rRNA sequencing. Functional profile analysis (PICRUTSt2) was used to predict metabolic functions associated with hydrocarbon degradation.

**Results:**

We found a large bacterial taxonomic diversity, including some genera reported as hydrocarbon-degraders. Analyses of the alpha and beta diversity did not detect significant differences between sites or seasons, suggesting that location, season, and the contamination level detected here do not represent determining factors in the structure of the microbial communities. PICRUTSt2 predicted 10 metabolic functions associated with hydrocarbon degradation. Most bacterial genera with potential hydrocarbon bioremediation activity were generalists likely capable of degrading different hydrocarbon compounds. The bacterial composition and diversity reported here represent an initial attempt to characterize sites with low levels of contamination. This information is crucial for understanding the impact of eventual rises in hydrocarbon pollution.

## Introduction

Contamination of marine ecosystems by hydrocarbons is caused by millions of tons of oil entering the sea each year through oil-pumping operations, high oil tanker traffic, refining activities, and the presence of submarine crude oil and refined product transport pipelines (Brooijmans, Pastink & Siezen, 2009; Daffonchio et al., 2012). Another large source of hydrocarbon pollution is oil spills. For instance, the Deepwater Horizon rig explosion released several hundred million liters of oil into the Gulf of Mexico (Crone & Tolstoy, 2010). Hydrocarbon pollution has a significant impact on coastal zones (Bociu et al., 2019; Han & Prabhakar Clement, 2018), resulting in devastating environmental damage with serious socioeconomic implications and highly toxic risks to humans (Chronopoulou et al., 2015; Crone & Tolstoy, 2010).

Crude oil is a complex of thousands of hydrocarbons (aliphatics and aromatics) and non-hydrocarbons (containing sulfur, nitrogen, oxygen and traces of various metals) (Mishra & Singh, 2012). Diverse chemical, physical and biological treatments for crude oil degradation have been deployed to reduce contamination. The choice of method depends on the type of pollutant and the characteristics of the contaminated site (Mahjoubi et al., 2018). Bioremediation is based on the metabolic capabilities of microorganisms and presents several advantages compared to classical remediation techniques, as an efficient, economic and environmentally friendly method (Bacosa, Suto & Inoue, 2012; Das & Chandran, 2011; Mahjoubi et al., 2018). For an effective bioremediation strategy, it is necessary to know the diversity of hydrocarbon-degrading bacteria and their hydrocarbon degradation potential (Mahjoubi et al., 2018). Bioremediation studies to date have reported a large number of bacteria as hydrocarbon-degraders (Brooijmans, Pastink & Siezen, 2009) and that saturated hydrocarbons have the highest biodegradation rates, followed by light aromatics, whereas high-molecular-weight aromatics and polar compounds exhibit extremely low rates of degradation (Leahy & Colwell, 1990).

Studies on sediment microbial communities suggest that the presence of hydrocarbons is one of the primary drivers structuring the microbial communities (Bacosa et al., 2018; Godoy-Lozano et al., 2018; Mason et al., 2014b). Bacterial diversity decreases with the increase in hydrocarbon content in sediment (Korlević et al., 2015; LaMontagne et al., 2004; Quero et al., 2015) and specific bacterial groups become dominant (Bacosa et al., 2018; Harayama, Kasai & Hara, 2004; Kessler et al., 2011; Mason et al., 2014b; Mason et al., 2014a; Mason et al., 2012; Valentine et al., 2010). There are numerous studies on the effect of hydrocarbon pollution in the microbial communities of sediments in the northern region the Gulf of Mexico (Bacosa, Liu & Erdner, 2015; Bacosa et al., 2018; Dong et al., 2019; Liu, Bacosa & Liu, 2017; Liu & Liu, 2013; Mason et al., 2014b; Mason et al., 2014a; Ramírez et al., 2020; Sánchez-Soto et al., 2018). However, information concerning the southern part of this area is lacking. Some studies in the southwestern Gulf of Mexico indicate that the shallow zone with the greatest aromatic hydrocarbon concentration was enriched with hydrocarbon-degrading bacteria (Godoy-Lozano et al., 2018). Research by the Mexican Petroleum Company (PEMEX) indicates that the Yucatan Peninsula is a potential oil reservoir, particularly in the coastal locality of Progreso. Despite the overwhelming consequences of its eventual exploitation, only one report on the marine ecosystems in the Yucatan Peninsula has been conducted (Reyes-Sosa, Apodaca-Hernández & Arena-Ortiz, 2018). The authors determined that the bacterial communities differed in sediment and water samples and established the presence of a high number of hydrocarbon-related genera in a location on the Yucatan shoreline (Reyes-Sosa, Apodaca-Hernández & Arena-Ortiz, 2018).

Nonetheless, for many years, Yucatan has maintained petroleum export activities to Central America through the Straits of Yucatan. Besides oil transport, these operations involve discharging and tanker washing (Botello et al., 2005). Coastal sites are prone to hydrocarbon contamination resulting from the marine current and winds that disperse the contaminants to shallow zones (Kappell et al., 2014). Despite the current risk, the hydrocarbon levels at coastal sites of the Yucatan Peninsula are unknown. The sole existing report on this subject focused on the differences in total petroleum hydrocarbons (TPH) during the rainy and dry seasons in Progreso (Valenzuela-Sanchez, Gold-Bouchot & Ceja-Moreno, 2005). Results obtained were above these limits, presenting TPH with higher weight molecular in rainy seasons.

The analysis of contamination levels accompanied by the microbial taxonomic baseline profile in a zone with hydrocarbon pollution is crucial for understanding its impact, and for evaluating and designing contingency plans and mitigation activities (Godoy-Lozano et al., 2018). In this regard, culture-independent techniques based on molecular tools such as high-throughput sequencing provide new insights into diversity and the functional potential of sediment microbial communities (Pandey, Chauhan & Jain, 2009).

In the current study, we describe and compare microbial communities in the sediment from two coastal sites with hydrocarbon pollution, Sisal and Progreso, during the dry and rainy seasons. We hypothesized that the presence of low levels of hydrocarbons at the coastal sites allows the existence of a microbial community with the functional potential for hydrocarbon degradation, most likely present at a low relative abundance. The knowledge generated in this work is important for understanding the impact of increased hydrocarbon pollution associated with an eventual massive petroleum exploitation.

## Materials & Methods

### Sediment collection

**Figure 1:**
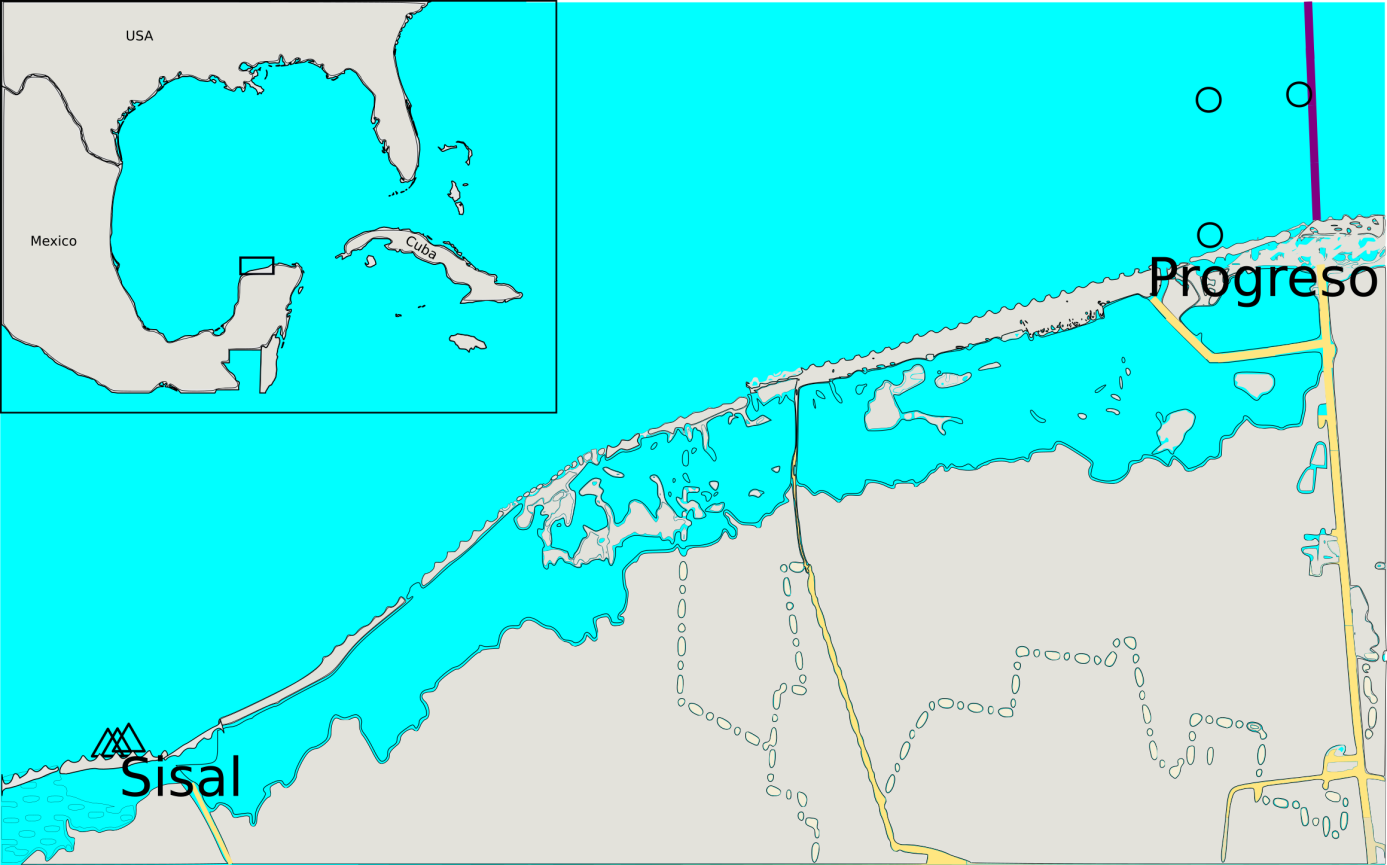
Collection site locations. Each site is indicated by a different symbol. The exact same locations were sampled during the dry and rainy seasons.

To study the dynamics of the sediment microbial communities in rainy and dry seasons, coastal sediment samples were collected in April-May 2013 (dry season) and June-August 2013 (rainy season) from Progreso and Sisal ports, Yucatan, Mexico. Both locations presented hydrocarbon pollution (Table S1, Fig. 1). Progreso and Sisal have urban, fisheries and port activities. Progreso is the main port in the Yucatan Peninsula, thus these activities are intense due to the tourism it receives (Herrera-Silveira et al., 2005). A total of 12 sediment samples were collected at Progreso (Pr) and Sisal (Si), which belonged to the dry (Dr) and rainy (Ra) seasons and were clustered in four sets (Tables S1 and S2). After sampling, samples were deposited in hermetic plastic bags and stored on ice upon collection and transportation to the lab and maintained frozen (−20 °C) until extraction was realized two years after.

### Physicochemical conditions and hydrocarbon quantification

Portable probes were used to determine pH, conductivity, temperature and redox of the sediment samples. A portion of sediments was frozen and processed to determine the total organic carbon (TOC) by the oxidation technique using potassium dichromate and acid medium (Brassard, 1997; Bricker, 1982). In addition, total nitrogen (N_2_T) and phosphorus (PiT) were quantified following the protocols by Parsons, Maita & Lali (1984) and Adams (1990).

Total petroleum hydrocarbons (TPH) were quantified by gravimetry using the methodology described by U.S. EPA (Fernández et al., 2006). Principal component analysis (PCA) of normalized data using a Euclidean distance matrix was performed to explore and visualize similarities among the samples based on environmental variables (depth, temperature, pH, the concentration of N2T, PiT, percentage of TOC) and TPH using the R package “vegan” (v1.17–2) (Oksanen et al., 2018). The statistical analyses used to assess the influence of the Site and Season factors on the physicochemical variables and TPH were a Shapiro–Wilk normality test and a two-way MANOVA (multivariate analysis of variance) test, both performed in R (R Core Team, 2019). A two-way analysis of variance (ANOVA at *p* < 0.05) was conducted to find the influence of the two factors (Site and Season) on each environmental variable using the “aov()” function of R (R Core Team, 2019).

### Genomic DNA (gDNA) extraction and generation of 16S rRNA amplicons

gDNA was extracted from 5 g of sediment following a standard extraction protocol (Zhou, Bruns & Tiedje, 1996) modified by Osborn et al. (2000) and García-Bautista et al. (2017). gDNA was precipitated with a solution of sodium acetate/isopropanol (0.3, 0.7 v/v) and washed with 70% ethanol. RNAse (10 mg/mL) was added to the final solution. DNA purification was carried out according to Lewis (2015).20 ng/µL of gDNA was used for the amplification of the V4 region of the 16S rRNA gene using primers 515F (5′-GTGCCAGCMGCCGCGGTA A -3′) and 806R (5′-GGACTACHVGGGTWTCTAAT3′). PCR Products were then quantified using the Quibit 2.0 fluorometer (Life Technologies) and Illumina adapters were ligated to construct sequencing libraries, which were sequenced using the paired-end method with an Illumina MiSeq (250-bp paired-end reads) at Research and Testing Laboratories (RTL) (Lubbock, TX, USA).

### Quality filters and sequence analysis.

Raw sequencing data was deposited in the Sequence Read Archive (SRA, http://www.ncbi.nlm.nih.gov/sra/) under accession number PRJNA631553.

Raw reads were overlapped to form contiguous reads using PEAR v.0.9.8 (Zhang et al., 2018) and de-multiplexed using a Phred score >Q30 by the QIIME v.1.9.1 pipeline (Caporaso et al., 2010). Sequences were clustered into operational taxonomic units (OTUs) and taxonomy was assigned using UCLUST v.1.2.22q (Edgar, 2010) based on 97% pairwise identity against the SILVA databases (SILVA v132) and Greengenes database (gg_13_8_otus). To increase taxonomic resolution, assigned and unassigned taxa were blasted against the NCBI database. Detection and removal of chimeric sequences were done with MOTHUR v.1.38.1 (Schloss et al., 2009). All OTUs belonging to the chloroplast and mitochondria, and sequences that did not have at least ten counts across all the samples were removed from the data set. The data set was rarefied based on the depth from the smallest library (8,879) using QIIME.

### Analysis of the sediment microbial community

We separated the 12 samples into sets (Table S2) to test the importance of the site and season in the structure of the sediment microbial community from two coastal sites, using the normalized OTU data. Alpha diversity (observed species, Shannon, and Chao indexes) estimated using the QIIME v.1.9.1 pipeline (Caporaso et al., 2010) and the differences among sediment sets were estimated with a Kruskal–Wallis test. Alpha diversity of the OTUs was visualized with boxplots using the “boxplot()” function in RStudio (RStudio Team, 2015). To test the importance of the site and season in each OTU and taxonomic group in the sediment microbial community, differences in the relative abundance of OTUs and taxa levels were estimated with a Kruskal–Wallis Rank Sum Test for all sediment sets using the Rhea Pipeline v.2.0 (Lagkouvardos et al., 2017). A rarefaction curve was computed directly using the “multiple_rarefaction” command of QIIME (Caporaso et al., 2010).

To measure taxonomic diversity among sediment samples (beta-diversity), non-metric multidimensional scaling (nMDS) ordination with a Bray-Curtis distance matrix was used (Bray & Curtis, 1957). The effect of the Site and Season factors was evaluated with a permutational multivariate analysis of variance (PERMANOVA) using a Bray-Curtis dissimilarities matrix previously calculated considering the relative abundances of OTUs in the samples. These analyses were performed using “vegan” for R (Oksanen et al., 2018). The significance threshold for the PERMANOVA was set at *p* < 0.001.

“Heat trees” of taxonomic diversity at the genus level from the 12 samples (normalized count = 8,879 reads) were developed using the R package “metacodeR” v.0.2.1 (Foster, Sharpton & Grunwald, 2017). In these plots, the node width and color indicate the number of reads assigned to each taxon. The most abundant OTUs and genera that belonged to bacteria and archaea were visualized in “barplot” in RStudio (RStudio Team, 2015).

PICRUST (the Phylogenetic Investigation of Communities by Reconstruction of Unobserved States) version 2 (Douglas et al., 2020) was used to predict functional capacities of the microbial communities in each sample. The enrichment analysis of pathways was performed based on the Kyoto Encyclopedia of Genes and Genomes (KEGG) database. The KEGG Orthologies (KOs) were classified at level 3, and the categories unrelated to bacterial metabolism and physiology were removed. To illustrate dissimilarities based on hydrocarbon degradation KOs, a heatmap was built using the R package “gplots” v.3.0.1 (Warnes et al., 2016). Differences in the predicted functions among sediment sets were tested using a Kruskal–Wallis test (we used the relative frequency, that means, the sum of the abundance of the KOs associated with a hydrocarbon function was divided by the abundance sum of all detected functions at level 3 for each sediment sample). Significant differences between pairs were determined using the pairwise Wilcoxon as a post hoc test with the Benjamini–Hochberg false discovery rate correction (Benjamini & Hochberg, 1995). Functional contributions of various taxa to different KOs were computed with “metagenome_contrib” command in PICRUSt2 (Douglas et al., 2020) and were visualized by “bar plots” in RStudio (RStudio Team, 2015).

## Results

### Environmental differences between sites and seasons

The physicochemical variables, and total petroleum hydrocarbons of sediment samples are shown in Table S1. There were no significant differences among the two sites and seasons studied here in terms of the physicochemical variables or TPH (MANOVA, *p* > 0.05). However, some variables such as temperature and TPH were significantly different between sites (ANOVA, *p* < 0.01), and among seasons and sites, respectively (ANOVA, *p* < 0.01) (Table S3). The temperature was higher in Sisal (with an average of 29.9 °C and 28.4 °C for the rainy and dry seasons respectively) than Progreso (with an average of 26.8 °C and 27 °C for the rainy and dry seasons respectively). The concentration of total petroleum hydrocarbons (TPH) was higher in Progreso (with an average of 157.5 µg/g and 92.5 µg/g for the rainy and dry seasons respectively) than Sisal (average of 80 µg/g and 66.7 µg/g for the rainy and dry seasons respectively). The principal component analysis based on the six environmental variables and TPH showed that the 12 sediment samples from Progreso and Sisal were not grouped according to site, season or sediment set (Fig. S1).

### Sediment microbial community.

From the 12 sediment samples, a total of 389,931 high-quality, filtered sequences and 4,323 OTUs were obtained, with a median of 18,566 sequences (ranging between 8879 and 84492) and 1,298 OTUs (ranging between 704 and 2,389) (Table S2). Rarefaction curves of the “observed OTUs” from the filtered OTUs table showed a saturating number of OTUs, indicating adequate sampling of 16S rRNA sequences for all samples (Fig. S2).

**Figure 2:**
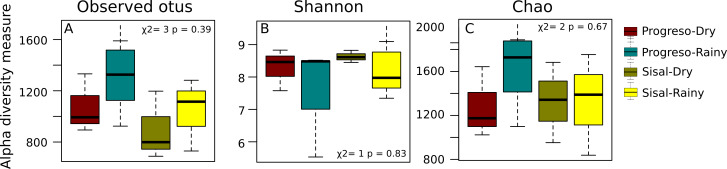
Distribution of alpha-diversity in the sediment microbial communities measured by the (A) OTUs, (B) Shannon and (C) Chao index.

**Figure 3:**
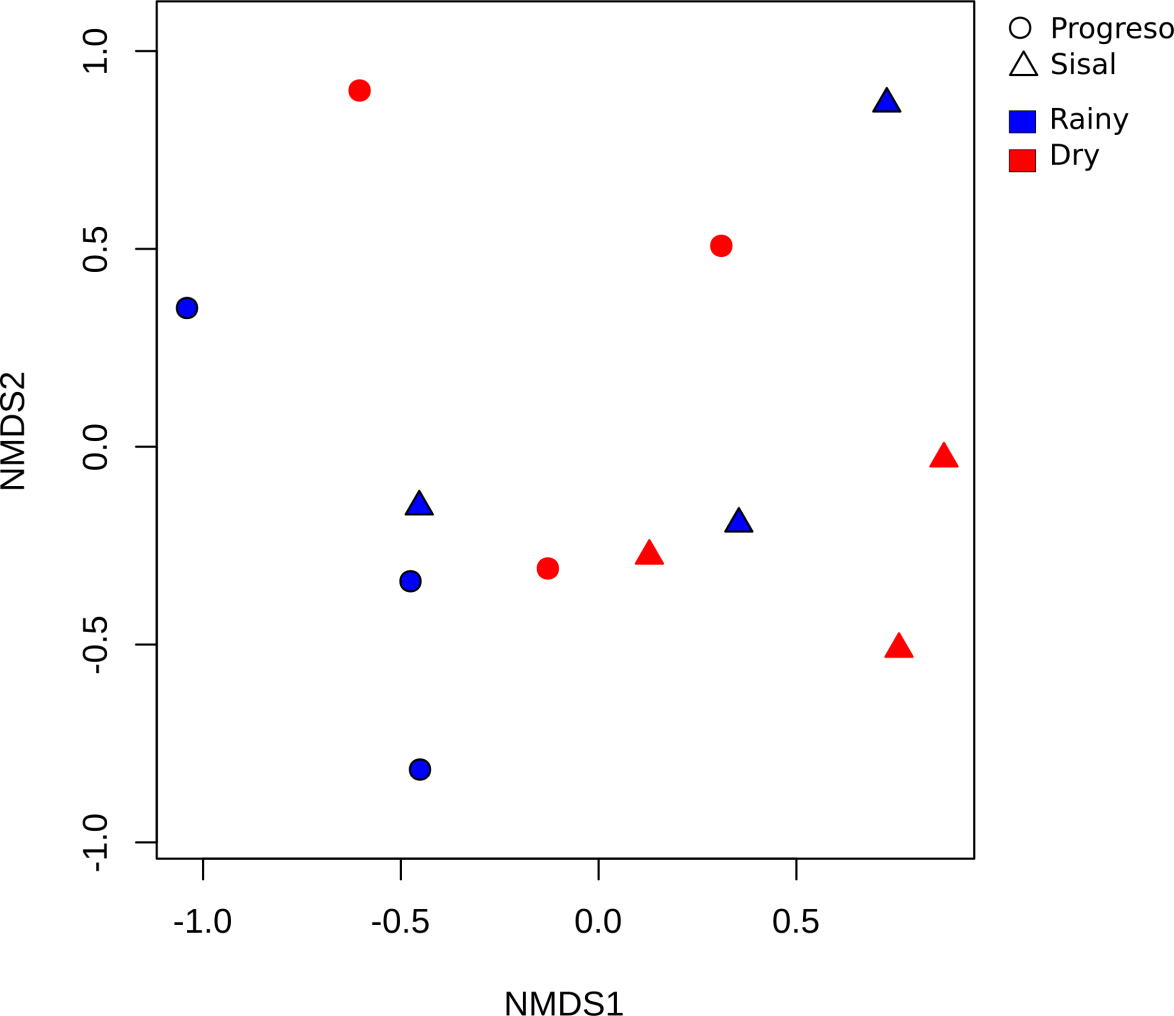
Beta diversity analysis of the sediment samples. Non-metric multidimensional scaling (nMDS) plot based on the Bray–Curtis distance of the 4,209 OTUs.

To measure the distribution of species diversity within the coastal sediment samples, alpha diversity metrics (observed species/OTUs, Shannon and Chao) were estimated. No significant differences in the alpha diversity were found between sediment sets (Fig. 2). nMDS analysis established that sediment microbial communities were not grouped according to the coastal site, season or sediment set based on their taxonomic abundance profiles (Fig. 3). This result was corroborated by permutational multivariate analysis of variance (PERMANOVA), in which the evaluation of the two factors (Site and Season) considered in the experimental design and their interaction revealed that none of them have an influence on sediment microbial communities (PERMANOVA, *p* > 0.05 for the two factors and interaction).

The analysis of the microbial diversity from sediment samples revealed that most OTUs and genera were shared between sediment sets (Fig. S3). Progreso-dry and Sisal-dry shared 1151 OTUs and 103 genera, representing 39% and 59% of the OTUs and genera present in both sediment sets. Progreso-rainy and Sisal-rainy shared 1429 OTUs and 102 genera, representing 40% and 55% of the OTUs and genera present in both sediment sets (Fig. S3B). Regarding the sediment sets by season, Progreso-dry and Progreso-rainy shared 1487 OTUs and 102 genera, representing 41% and 61% of the OTUs and genera present in both sediment sets (Fig. S3A). The Sisal-dry and Sisal-rainy sets shared 1275 OTUs and 116 genera, representing 46% and 64% of the OTUs and genera present in both sediment sets (Fig. S3A). 698 OTUs and 76 genera were shared by all the sediment sets. These results suggest that the sites and seasons shared a similar microbiome composition. Similar results were found in the analysis of the abundance of each OTU and taxon, which showed no significant differences between sediment sets.

**Figure 4:**
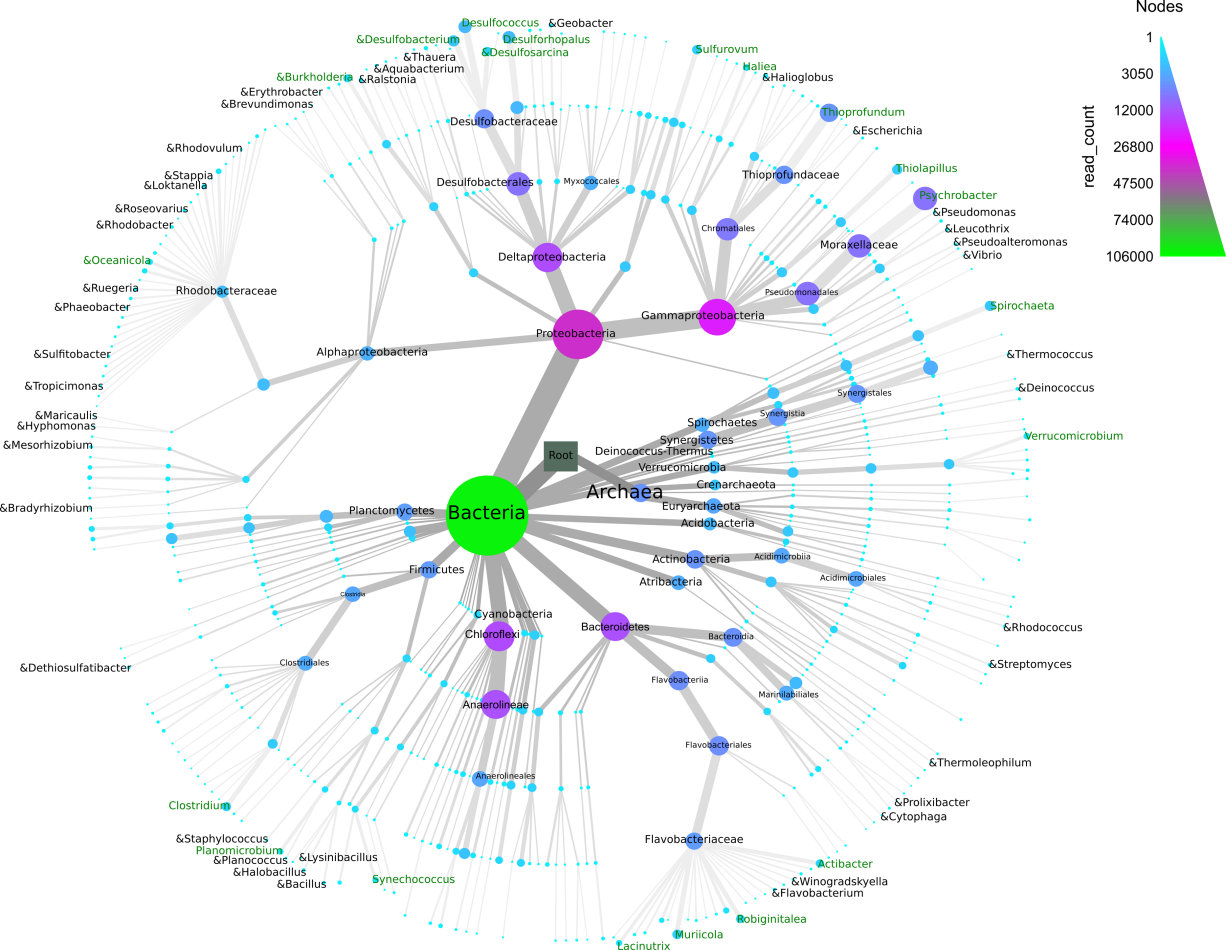
Taxonomic diversity and abundance of Bacteria and Archaea found in the sediment samples from Progreso and Sisal. The “&” symbol indicates the bacterial genera that have been reported as hydrocarbon-degraders. The node width and color indicate the number 0.

The normalized OTU data (normalized count = 8,879 reads) were assigned to different taxonomic levels, yielding 4,209 OTUs, 212 genera, 143 families, 118 orders, 65 classes, and 40 phyla. A small number of OTUs and reads were identified as Archaea (451 OTUs/4% of total reads). At the phylum level, members of Proteobacteria, Chloroflexi, Bacteroidetes, Actinobacteria and Synergistetes dominated with 35.7%, 12.4%, 11.5%, 6.2% and 4.5% respectively (Fig. 4). All other phyla and unclassified phyla contributed with less than 24% and 6% of total sequences, respectively. The most common classes were Gammaproteobacteria (19%), Deltaproteobacteria (12%), Anaerolineae (11.6%), Flavobacteriia (4.9%) and Bacteroidia (4.6%). Dominant families were Moraxellaceae (7.4%), Desulfobacteraceae (5%), Thioprofundaceae (4.4%), Flavobacteriaceae (3.8%) and Dethiosulfovibrionaceae (2.6%) (Fig. 4).

A total of 212 genera were identified (Fig. 4), and the 20 most abundant were evenly distributed among the samples. A similar trend was observed for the 20 most abundant OTUs (Figs. S4A and S4B). The five most abundant genera (relative abundance of total reads) in the 12 sediment samples included *Psychrobacter* (7%), *Thioprofundum* (4%), *Desulfococcus* (2%), *Desulforhopalus* (2%) and *Desulfobacterium* (1%) (Table S2 and Fig. 4). Six genera belonging to Archaea (*Methanogenium, Nitrosopumilus, Methanolobus, Methanosalsum, Methanococcoides* and *Methanobrevibacter*) were found in the coastal sediment samples (Fig. 4). Some bacterial genera found in the sediment microbial communities have been reported as hydrocarbon-degraders, although their relative abundance was low (Table S4).

98 KOs related to 10 metabolic functions associated with hydrocarbon degradation were found among the sediment microbial communities, which represented 0.7% of the total potential function (total related readings in the functional prediction analysis of 6,834 KOs detected in our study) (Table S5).

**Figure 5:**
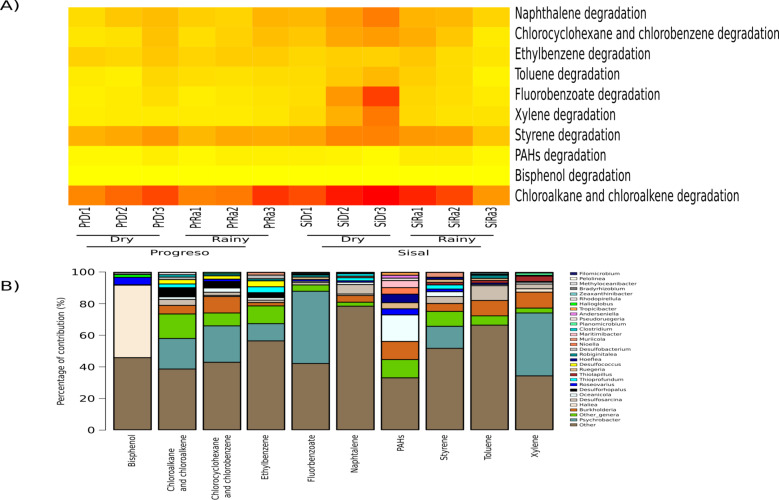
KEGG categories (at level 3) associated with hydrocarbon-degradation found in the sediment microbial communities. (A) Heat map of 11 categories, each column corresponds to a sediment sample and each row corresponds to a specific category. (B) Top 10 most important contributors to each metabolic function. The taxa associated with the functions are represented by different colors and “Other” represents the OTUs that were not assigned to the genus level.

The most abundant metabolic functions were chloroalkane and chloroalkene degradation, followed by styrene degradation (Fig. 5A). These metabolic capabilities were compared between localities and seasons based on the relative abundance of the specific KOs sum predicted by PICRUSt. Only xylene degradation was significantly more abundant in the two sediment sets from Sisal (rainy and dry seasons) than in Progreso (X2 = 8.9, *p*-value = 0.03). However, in the paired test, no pair was significantly different.

PICRUSt analyses of the taxa contributing to the 10 metabolic functions in degradation, determined that genera such as *Oceanicola, Burkholderia, Desulfosarcina, Roseovarius* and *Psychrobacter* contributed to several hydrocarbon degradation functions (Fig. 5B); in particular, *Haliea* (46%) (percentage of contribution) in bisphenol degradation, and *Psychrobacter* in chloroalkane and chloroalkene (19%), chlorocyclohexane and chlorobenzene (23%), ethylbenzene (11%), fluorobenzoate (46%), styrene (14%) and xylene (40%) degradation. For toluene degradation, the main contributor was *Burkholderia* (28%). The genus *Oceanicola* plays a major role in polycyclic aromatic hydrocarbon degradation (17%) (Fig. 5B). In naphthalene degradation (24%), the genus *Desulfosarcina* (6%) was the main contributor.

## Discussion

### Sediment microbial communities at sites with low hydrocarbon pollution

The main finding of this study indicated that regardless of the presence of hydrocarbons detected at the two sites along the Yucatan coastline (significantly larger in Progreso), the levels of contamination do not represent a stress factor that could lead to changes in the microbial community structure of the sediments in our study area, as has been reported in other coastal areas (Engel & Gupta, 2014; Quero et al., 2015).

The average level of hydrocarbon pollution determined in this study for the four sediment sets was 99 µg/g, which is slightly higher than the permissible level in coastal sediments (70 µg/g) (UNESCO, 1976). However, in Progreso an increase in hydrocarbon pollution has been observed over time. Hydrocarbon analyses from 2005 indicated that the mean TPH value was 16.62 µg/g and 23.25 µg/g for rainy and dry seasons respectively, indicating an increase in hydrocarbon content in eight years (Valenzuela-Sanchez, Gold-Bouchot & Ceja-Moreno, 2005).

Some authors have suggested that microbial community analysis associated with sites showing low levels of contamination is crucial for understanding the impact of an increase in hydrocarbon pollution caused by eventual oil spills (Godoy-Lozano et al., 2018) and/or by anthropogenic activities (Valenzuela-Sanchez, Gold-Bouchot & Ceja-Moreno, 2005). The alpha-diversity analysis showed that the sediment microbial community did not differ between sites or seasons, and no individual OTU or taxa were more abundant in any sediment set.

Studies have reported a decrease in microbial richness associated with increased chemical contamination in sediment (Quero et al., 2015), ranging from 200 to 1000+ bacterial genera depending on the level of hydrocarbon pollution (Korlević et al., 2015). However, these variables did not shape community diversity in Progreso or Sisal for either season. We do not exclude the idea that other chemical pollutants and eutrophication by human activities at the port sites could have an important influence on the microbial community structure or some taxa, as has been reported in other coastal areas (Chen et al., 2019; Quero et al., 2015; Sun et al., 2013).

Hydrocarbons have been considered a primary driver structuring the microbial community in marine sediments (Bacosa et al., 2018; Godoy-Lozano et al., 2018; Mason et al., 2014b; Quero et al., 2015; Wang et al., 2016). However, we found no differences in the microbial communities from two sites along the coastline of Yucatan with differences in hydrocarbon levels. We suggest that if the two localities had more contrasting characteristics, such as the physicochemical conditions, environment types and a larger concentration of hydrocarbon contaminants, differences in the microbial communities may be evident. Quero et al. (2015) reported differences in community composition and the formation of two clusters corresponding to two coastal sites with significant differences in the environmental variables and levels of hydrocarbon contamination. Differences in sediment microbial communities have been detected between contrasting sites, such as shallow and deep marine zones (Godoy-Lozano et al., 2018; Sánchez-Soto et al., 2018), water versus sediment samples (Reyes-Sosa, Apodaca-Hernández & Arena-Ortiz, 2018; Ul-Hasan et al., 2019), sediment from different marine ecosystems (lagoon, sea, wetlands, freshwater upwelling) (Reyes-Sosa, Apodaca-Hernández & Arena-Ortiz, 2018), and in microcosm experiments with hydrocarbons added in different concentrations (Bacosa et al., 2018; Wang et al., 2016). We found no clustering by site or season, with a high intra-variation between samples. A similar pattern was observed by Sánchez-Soto et al. (2018), which reported intra-variation among shallow sediment samples versus deep samples from the northwestern Gulf of Mexico, which were clustered.

The presence of hydrocarbon-degrading microbial populations in marine sediments with different levels of hydrocarbon pollution has been reported in several marine ecosystems, such as beaches (Engel & Gupta, 2014), coastal areas (Godoy-Lozano et al., 2018; Korlević et al., 2015; Miettinen et al., 2019; Quero et al., 2015; Sánchez-Soto et al., 2018), deep zones (Godoy-Lozano et al., 2018; Sánchez-Soto et al., 2018) and hydrocarbon-enriched microcosms using microorganisms or sediments from marine sources (Bacosa et al., 2018; Wang et al., 2016). An increase in the abundance of hydrocarbon-degrading bacteria has been reported as an indicator of high levels of hydrocarbon contamination in sediments from backshore areas (Engel & Gupta, 2014; Kostka et al., 2011), and in microcosm experiments with hydrocarbon addition (Bacosa et al., 2018; Liu, Bacosa & Liu, 2017; Wang et al., 2016). We suggest that the microbial composition and diversity reported in our study are different from those reported in other studies with higher levels of hydrocarbon pollution. The *Colwellia* and *Cycloclasticus* genera were dominant in the bacterial communities in the deepwater oil plume during the oil spill in the Gulf of Mexico (Kessler et al., 2011; Mason et al., 2012; Valentine et al., 2010) and were absent in our data. These genera have been associated with or enriched in heavily oil-impacted sediments (Mason et al., 2014b; Mason et al., 2014a) and have high potential as hydrocarbon-degraders (Bacosa et al., 2018; Harayama, Kasai & Hara, 2004). Other genera absent in our data were *Alcanivorax, Oleiphilus* and *Oleispira*, which are widely acknowledged as “professional hydrocarbonoclastic bacteria” using hydrocarbons as a primary carbon source (Harayama, Kasai & Hara, 2004). Bacosa et al. (2018) reported an increase in the relative abundance of *Alcanivorax*, *Bacteriovorax*, *Pheaeobacter* and *Octadecabacter* with the addition of oil to marine sediments (Bacosa et al., 2018). These genera were found to be absent or found at low abundance in our data. The degradation of a particular hydrocarbon has been positively correlated with the cell density of some bacterial groups, including hydrocarbon-degrading bacteria (Liu, Bacosa & Liu, 2017). Also, hydrocarbon-degrading bacteria showed low abundance or were undetectable before hydrocarbon pollution, but dominate after oil contamination (Yakimov, Timmis & Golyshin, 2007). Overall, of the 20 most abundant genera in this study, only *Desulfobacterium, Desulfosarcina*, *Burkholderia* and *Oceanicola* have been reported as hydrocarbon-degrading bacteria (Chikere, Okpokwasili & Chikere, 2011; Lee et al., 2016), and most microbial genera with potential as hydrocarbon degraders found in this study had low relative abundance.

In contrast to the previously mentioned genera, the genus *Thioprofundum* was one of the most abundant in our sediment samples. This genus is a known sulfur oxidizer (Takai et al., 2009), and might play an important role in the cycling of sulfur in coastal sediments. Godoy-Lozano et al. (2018) reported an increase in the abundance of this genera at sites with low hydrocarbon concentrations compared with sediments disturbed by an oil spill. These authors reported that their results are comparable to those in other studies that failed to recover this genus in oil spill-perturbed sediments, suggesting that the genus *Thioprofundum* may act as a biomarker of healthy marine sediments. The presence of the genus *Desulfococcus* in this and other studies in shallow sediments conducted at sites with low contamination that eventually could be subjected to oil extraction activities (Sánchez-Soto et al., 2018) may indicate that this genus could represent another biomarker of healthy marine sediments.

### Functional potential from the sediment microbial community

The results of the PICRUSt2 analysis showed that the sediment microbial communities in our samples carry 10 metabolic functions associated with hydrocarbon degradation. These predicted functions represented a low percentage of the total potential functions, which supports the low levels of hydrocarbons found in the TPH analysis and the fact that the sediments of our study area are not heavily polluted.

The predicted hydrocarbon degradation found in our study has been reported in different marine environments characterized by not having a direct source of high levels of hydrocarbon pollution (Reyes-Sosa, Apodaca-Hernández & Arena-Ortiz, 2018). In a PICRUSt analysis on shallow and deep microbial communities from the northwestern Gulf of Mexico, Sánchez-Soto et al. (2018) reported the functional potential to degrade aromatic compounds, such as ethylbenzene, chlorocyclohexane and fluorobenzoate. These authors suggested that these metabolic functions could contribute to bioremediation in the case of hydrocarbon contamination due to forthcoming oil exploration and extraction activities. Some microcosm experiments with hydrocarbon addition showed that the functions predicted by PICRUSt were similar to those obtained in their hydrocarbon degradation experiments (Liu, Bacosa & Liu, 2017; Wang et al., 2016).

Hydrocarbon biodegradation and the development of hydrocarbon-degrading microbial communities depend on environmental conditions (Deeb & Alvarez-Cohen, 1999; Li et al., 2012; Liu, Bacosa & Liu, 2017) and the nature of the petroleum (Das & Chandran, 2011; Deeb & Alvarez-Cohen, 1999). Hydrocarbons differ in their susceptibility to microbial attacks. Saturated (aliphatic) exhibit higher rates of biodegradation followed by aromatic light compounds (Leahy & Colwell, 1990). These two types of hydrocarbons predominate in our PICRUSt analysis. Chloroalkane, chloroalkene and styrene degradation pathways were represented at relatively high frequency in the samples. It would be reasonable to think that the enrichment in degradation pathways of aromatic and chlorinated hydrocarbon correlates with the increase of such compounds in these areas. In that sense, n-alkanes are the main constituents of petroleum and its refined products (Mardanov et al., 2009) and styrene has been identified as a major soil contaminant, where it is believed to be immobilized in part due to polymerization (Timmis, 2010). Conversely, low detection of the degradation of other hydrocarbons such as PAHs could be associated with their hydrophobic nature, which permits their accumulation in fine-grained sediment (Valenzuela-Sanchez, Gold-Bouchot & Ceja-Moreno, 2005), and our texture data showed the presence of coarse grains due to the majority percentage of sand (Table S1).

As in other studies (Bacosa et al., 2018; Engel & Gupta, 2014; Korlević et al., 2015; Miettinen et al., 2019; Quero et al., 2015), the analysis of taxa contributing to the 10 metabolic functions through PICRUSt predictions indicated a large genera diversity associated with hydrocarbon degradation. A larger microbial diversity has shown advantages for hydrocarbon degradation over a single functional bacterium (Wang et al., 2008), and generalist bacteria are capable of degrading structurally different hydrocarbons, such as n-alkanes, branched alkanes and PAHs (Wang et al., 2016). We found that several bacteria genera have the potential to contribute to degrading different components of crude oil (PICRUSt analysis).

For example, the genus *Burkholderia* had the potential for contribute with functions associated with the degradation of nine hydrocarbons, mainly toluene. This genus has been associated with the degradation of xylene and decane (Bacosa, Suto & Inoue, 2012), and its genome possesses putative gene clusters for biodegradation of various monocyclic aromatic hydrocarbons (MAHs), including benzoate, toluene, and xylene (Lee et al., 2016).

The genus *Rhodovulum* had the potential for contribute in eight metabolic functions,included naphthalene degradation, where was the main contributor. Naphthalene degradation activities have been previously reported for this genus (Teramoto et al., 2010). Also, genomes from strains of *Rhodovulum* encode enzymes associated with the degradation of naphthalene (naphthalene 1,2-dioxygenase) and other hydrocarbons found in the PICRUSt analysis, such as polycyclic aromatic hydrocarbons (protocatechuate 3,4-dioxygenase) and fluorobenzoate (catechol 1,2-dioxygenase) (Brown et al., 2015).

Other genera found in this study that had the potential for contributed to hydrocarbon degradation, were Halioglobus, Loktanella, Pseudoalteromonas, Pseudomonas, and Vibrio, which had differential expression in oil-degrading (alkanes and aromatic hydrocarbons) genes in microcosm experiments (Tremblay et al., 2019).

The genera *Oceanicola* and *Desulfosarcina* had the potential as main contributors in 10 and nine metabolic functions, respectively. However, to date only naphthalene, toluene and xylene degradation have been experimentally confirmed (Alain et al., 2012; Chikere, Okpokwasili & Chikere, 2011; Hassanshahian & Boroujeni, 2016; Weelink, Van Eekert & Stams, 2010).

The formation of bacterial consortiums is a determining factor for hydrocarbon degradation. Some bacterial genera found in this study could constitute consortiums to degrade different kinds of polycyclic aromatic hydrocarbons (PAHs) (Bacosa, Suto & Inoue, 2012; Cameotra & Singh, 2008; Li et al., 2012; Wang et al., 2008). For instance, *Pseudomonas* and *Rhodococcus* have the ability to form a microbial consortium to effectively degrade n-hexadecane by biosurfactant production (Cameotra & Singh, 2008). *Rhodococcus* was shown to utilize benzene, toluene and ethylbenzene as primary carbon and energy sources (Deeb & Alvarez-Cohen, 1999), while *Pseudomonas* can degrade aromatic hydrocarbons and has catechol 2,3-dioxygenase genes (Brusa et al., 2001), which are part of the metabolic pathways in the degradation of some hydrocarbons found in our PICRUSt analysis, such as styrene, xylene, chlorocyclohexane and chlorobenzene. In this study the functional potential was predicted using 16S rRNA gene amplicons, however, we are aware that this DNA-based method does not discriminate between DNA from live (dormant cells, growing or non-growing metabolically active cells) and dead microbial cells (Li et al., 2017), therefore, caution should be exercised in interpreting the activity of predicted functions and their possible contributors. Considering these limitations, we suggest that future research in sediment microbial communities should combine high-throughput sequencing and methods that allow detecting live bacterial cells as Propidium-monoazide (PMA) and RNA-based sequencing methodologies, which have been used in several bacterial microbiota studies (Li et al., 2017; De Vrieze et al., 2018; Wurm et al., 2018).

## Conclusions

The present study represents an attempt to characterize sediment microbial communities in environments with low hydrocarbon pollution. Degradation profiles of different bacteria are crucial for the selection of the ideal microbes for future hydrocarbon bioremediation. Considering the limited 16S rRNA information, this study was not able to provide a complete picture of the mechanism for hydrocarbon biodegradation by the sediment microbial communities. Further research should focus on the function (shotgun metagenomics) and gene expression patterns (metatranscriptomics) of the sediment microbial communities associated with hydrocarbon pollution in microcosms and in situ.

##  Supplemental Information

10.7717/peerj.10339/supp-1 Supplemental Information 1 Sediment physicochemical variables

10.7717/peerj.10339/supp-2 Supplemental Information 2 Number of reads, OTUs and genera as well as the most abundant genera obtained from each sediment sample

10.7717/peerj.10339/supp-3 Supplemental Information 3 Environmental and physicochemical variables analysis of variance (ANOVA) Significant differences are indicated by an asterisk.

10.7717/peerj.10339/supp-4 Supplemental Information 4 Relative abundance of bacterial genera found in the current study and reported as hydrocarbon-degraders

10.7717/peerj.10339/supp-5 Supplemental Information 5 Table S5 KEGG ortholog groups (KOs) associated with hydrocarbon degradation used in the functional comparisons between sediment sets at the gene level. The gene, KEGG description, enzyme class and function are shown.

10.7717/peerj.10339/supp-6 Supplemental Information 6 Principal component analysis (PCA) based on the six environmental variables and total petroleum hydrocarbons PCA shows that the sediment samples were not grouped according to the site or season.

10.7717/peerj.10339/supp-7 Supplemental Information 7 Rarefaction curves of the OTUs observed in the 12 sediment samples Each color represents a sediment sample.

10.7717/peerj.10339/supp-8 Supplemental Information 8 Venn diagram of the shared OTUs (in black) and genera (in italic) among the sediment sets

10.7717/peerj.10339/supp-9 Supplemental Information 9 Taxonomic composition of the 20 most abundant bacterial OTUs (A) and genera (B) in the sediment sets The relative abundance of the bacterial genera is represented by different colors. The taxonomic level is indicated by a letter: p = phylum, c = class, o = order, f = family and g = genus.

## Additional Information and Declarations

### Data Availability

The following information was supplied regarding data availability: Raw sequence reads are available at the Sequence Read Archive: PRJNA631553.
