# Supplementary figures and images for: Characterization of sediment microbial communities at two sites with low hydrocarbon pollution in the southeast Gulf of Mexico
Source: PeerJ. 2020 Dec 8;8:e10339. doi: 10.7717/peerj.10339 (PMC7731659; doi:10.7717/peerj.10339)

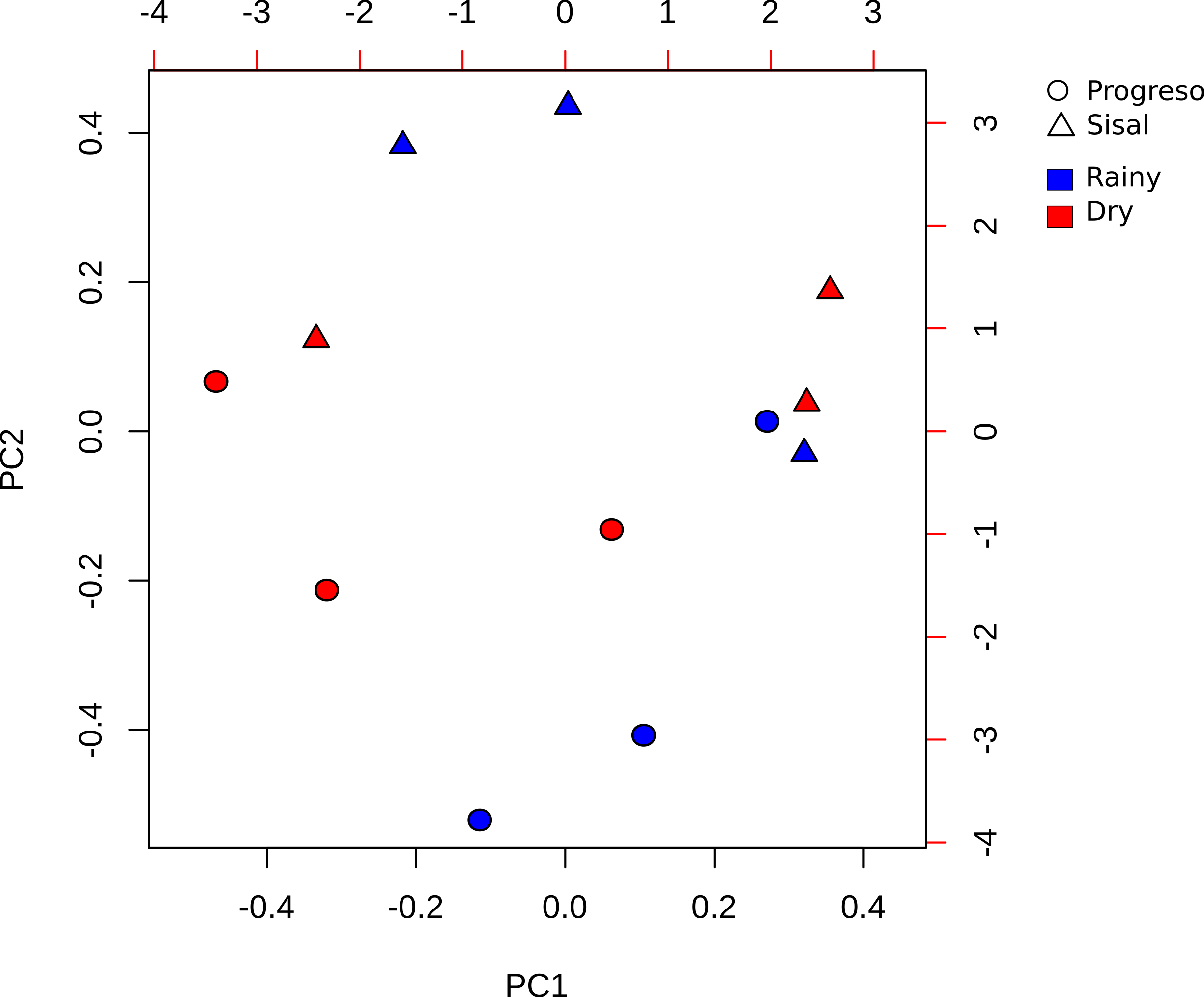

Supplement: Supplemental Information 6 — PCA shows that the sediment samples were not grouped according to the site or season. [file peerj-08-10339-s006.png]

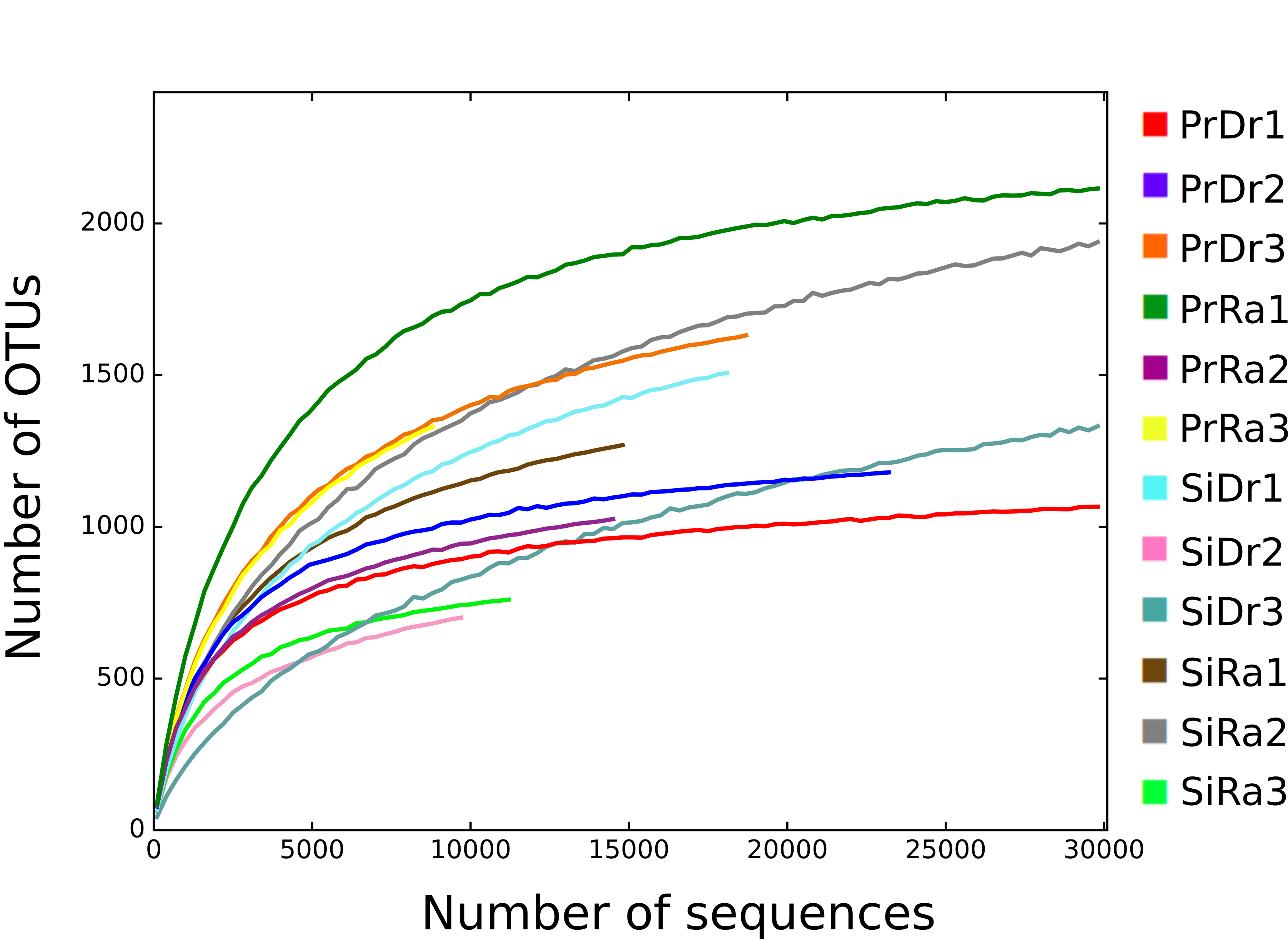

Supplement: Supplemental Information 7 — Each color represents a sediment sample. [file peerj-08-10339-s007.png]

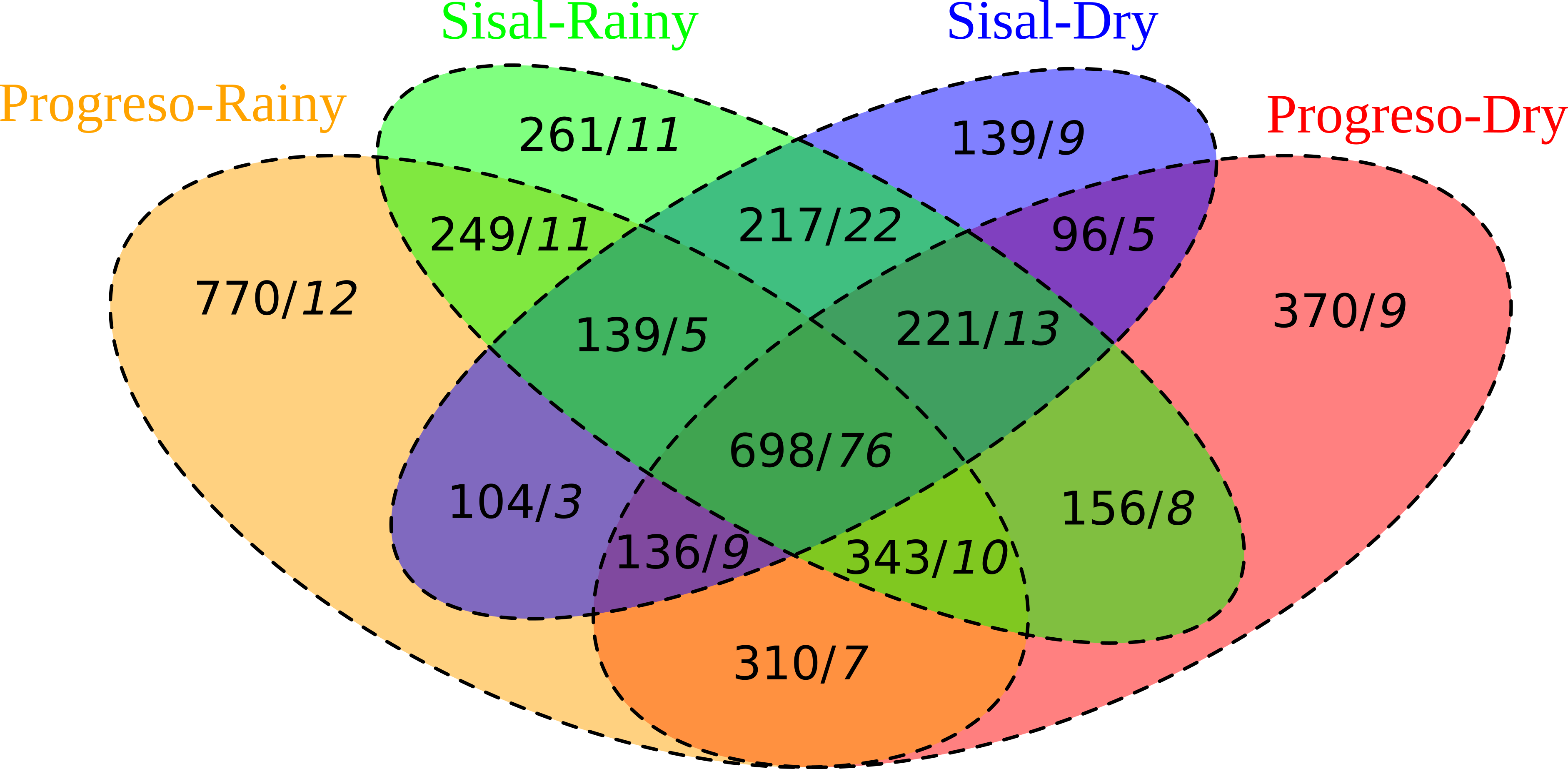

Supplement: Supplemental Information 8 [file peerj-08-10339-s008.png]
